# Persistent negative symptoms in the EULAST cohort: impact on functional outcome

**DOI:** 10.1038/s41537-026-00739-w

**Published:** 2026-03-02

**Authors:** Luigi Giuliani, Pasquale Pezzella, Giulia M. Giordano, Andrea Perrottelli, Armida Mucci, Paola Bucci, Istvan Bitter, Celso Arango, Jurjen J. Luykx, Covadonga M. Díaz-Caneja, Bjørn H. Ebdrup, Michael Davidson, Jari Tiihonen, Inge Winter-van Rossum, Silvana Galderisi

**Affiliations:** 1 University of Campania “Luigi Vanvitelli”, Naples, Italy; 2 Department of Psychiatry and Psychotherapy, Semmelweis University, Budapest, Hungary; 3 Department of Psychiatry. Hospital La Paz. IDIPAZ. Universidad Autonóma de Madrid. CIBERSAM, Madrid, Spain; 4 Department of Child and Adolescent Psychiatry, Institute of Psychiatry and Mental Health, Hospital General Universitario Gregorio Marañón, School of Medicine, Universidad Complutense, IiSGM, CIBERSAM, Instituto de Salud Carlos III, Madrid, Spain; 5 Department of Psychiatry, Amsterdam University Medical Center, Amsterdam, the Netherlands; 6 Department of Psychiatry and Neuropsychology, School for Mental Health and Neuroscience, Maastricht University Medical Center, Maastricht, the Netherlands; 7 Center for Neuropsychiatric Schizophrenia Research (CNSR), Mental Health Center, Glostrup, Copenhagen University Hospital – Mental Health Services CPH, Copenhagen, Denmark; 8 Department of Clinical Medicine, Faculty of Health and Medical Sciences, University of Copenhagen, Copenhagen, Denmark; 9 Department Basic and Clinical Sciences, Nicosia University Medical School, Nicosia, Cyprus; 10 Karolinska Institutet, Department of Clinical Neuroscience and Centre for Psychiatry Research, Stockholm Health Care Services, Region Stockholm, Stockholm, Sweden; University of Eastern Finland, Department of Forensic Psychiatry, Niuvanniemi Hospital, Kuopio, Finland; 11 Department of Psychiatry, UMC Brain Center, University Medical Center Utrecht, Utrecht University, Utrecht, Netherlands; Department of Psychiatry, Icahn School of Medicine at Mount Sinai, New York, NY USA

**Keywords:** Schizophrenia, Psychosis

## Abstract

Persistent negative symptoms (PNS), defined as negative symptoms of at least moderate severity that endure over time and are not attributable to other psychopathological dimensions such as depression or parkinsonism, have been associated with poor functional outcomes in schizophrenia, in both chronic stages and in the early phases of the disorder. This post-hoc analysis of a large cohort of schizophrenia spectrum disorder patients within their first 7 years of illness, enrolled in the European Long-acting Antipsychotics in Schizophrenia Trial (EULAST), aimed to: 1) confirm prior findings about the prevalence and clinical impact of unconfounded persistent negative symptoms (PNS) on dropout rates and psychosocial functioning after 12 and 18 months of treatment; and 2) explore the prevalence of enduring negative symptoms (E-NS), defined as persistent negative symptoms either confounded or unconfounded by depression or parkinsonism, and their influence on functional outcome. At week 0, 60.6% of patients exhibited at least one negative symptom of moderate severity. Among them, 42.8% met criteria for unconfounded negative symptoms. After 1 year, the frequency of PNS and E-NS was 7.9% and 15%, respectively, with a prospective consistency around 32%. PNS subjects had similar levels of functioning at week 0 (*d* = −0.179, *p* = 0.194), but worse functioning after 12 (*d* = −0.697, *p* = 0.028) and 18 (*d* = -0.676, *p* = 0.024) months of treatment, as compared to those with negative symptoms of similar severity at baseline that did not persist (non-persistent negative symptoms, N-PNS). No difference among groups was observed in drop-out rates. The comparison between the E-NS and N-PNS groups revealed the same functional outcome differences observed in the PNS versus N-PNS comparison. Our findings confirm that long-term persistence of negative symptoms, both primary and secondary, contributes to poor functional outcome. Future research should focus on identifying predictors of symptom persistence to guide the development of targeted interventions aimed at improving long-term functional outcomes in this patient population.

## Introduction

The presence of negative symptoms in schizophrenia is an important unmet therapeutic need^1–3^. Negative symptoms can be detected during the early stages of the disorder, even before the onset of psychotic symptoms^3–10^ are related to impaired premorbid functioning and show an elevated stability throughout the course of the illness^11–14^. They have a severe impact on functional outcomes and quality of life^15–20^ and might predict early treatment discontinuation and inadequate treatment response^13,21–25^. However, currently available treatments are often ineffective, especially for primary and/or persistent negative symptoms, and no pharmacological intervention has yet received regulatory approval with an indication for these symptoms^21,26^.

It is widely recognized that negative symptoms of schizophrenia represent a heterogeneous clinical construct, including symptoms with diverse clinical expression and courses, which likely stem from different pathways and may necessitate different treatment approaches^27–32^. Multiple efforts have been made to reduce the heterogeneity of negative symptoms, not only in research settings but also in clinical contexts. Among proposed approaches, the identification of Persistent Negative Symptoms (PNS)^2,33^ is considered a promising strategy for clinical trials evaluating treatment efficacy in this psychopathological domain^34–37^. This construct, by definition, encompasses negative symptoms that, notwithstanding adequate treatment with antipsychotic drugs, persist over time, even during periods of clinical stability. In early stages of schizophrenia, persistence of negative symptoms, especially of primary ones, which are intrinsic to the illness and not secondary to factors such as depression, parkinsonism, or positive symptoms, predicts worse clinical and functional outcome^12,13^, lower quality of life^38,39^, as well as scarce treatment adherence^40^. According to the original definition, PNS should present at least a moderate severity, should not be secondary to previously mentioned conditions as ascertained by established and validated rating scales, and should be present for at least 6 months ^2,33^.

Prompt recognition and accurate evaluation of PNS since the early phases of the illness is an important and necessary step to promote the development of adequate treatment strategies.

Previous research conducted in individuals experiencing their first episode of psychosis has indicated a prevalence of PNS ranging from 4 to 41%^5,12,13,41–50^. Variations in prevalence rates stem primarily from the different criteria employed to define PNS across studies, including diagnostic criteria, duration of follow-up, and control for potential secondary sources of negative symptoms.

Several studies highlight the clinical relevance of PNS as an important determinant of functional outcome in schizophrenia, especially in the earliest stages of the illness^3,37,51^. In recent-onset cohorts, patients with PNS exhibit poorer psychosocial functioning, lower remission rates, and increased likelihood of treatment resistance over time^12^. The EUFEST study showed that patients with PNS, compared to those with negative symptoms of similar severity at baseline that did not persist (non-persistent negative symptoms, N-PNS), had significantly worse global functioning and greater treatment discontinuation rates after one year, despite similar baseline severity of negative symptoms^13^. Additional studies have linked PNS to deficits in social and occupational engagement, lower quality of life, and reduced real-world functioning ^5,19,37,52^.

Taking into account this evidence, in the present post-hoc analysis, we aimed to confirm previous findings in an independent cohort of recent-onset schizophrenia spectrum patients with an illness duration of up to 7 years, enrolled in the European Long-acting Antipsychotics in Schizophrenia Trial (EULAST) study. We explored differences in functional outcome and drop-out rates between study participants with PNS—i.e., persistent negative symptoms unconfounded by depression or parkinsonism—and those who had a comparable severity of negative symptoms at baseline, whose negative symptoms did not persist at 1 year follow-up (N-PNS) (Table 1). Additionally, we compared individuals with enduring negative symptoms (E-NS)—i.e., persistent negative symptoms, either unconfounded or confounded by depression or parkinsonism (Table 1)—with N-PNS participants. We hypothesized that individuals with PNS and E-NS would have a higher drop-out rate and worse psychosocial outcome than N-PNS participants.

**Table 1:** Operational criteria for negative symptom subgroups.

Negative symptoms subgroups	Criteria
Unconfounded negative symptoms	Presence of at least one core negative symptom of moderate severity at baseline (week 0), together with the absence of clinically significant depression (PANSS G6 ≤ 3) and clinically significant parkinsonism (according to SHRS criteria).
Confounded negative symptoms	Presence of at least one core negative symptom of moderate severity at baseline (week 0), but with clinically significant depression (PANSS G6 ≥ 4) and/or clinically significant parkinsonism (according to SHRS criteria).
Persistent negative symptoms	Presence of unconfounded negative symptoms at baseline (week 0) that persisted after one year without becoming confounded by depression or parkinsonism.
Non-persistent negative symptoms	Presence of at least one core negative symptom of moderate severity at baseline (week 0) that did not persist after 1 year.
Enduring negative symptoms	Presence of at least one core negative symptom of moderate severity at baseline (week 0) that persisted after 1 year, regardless of whether symptoms were confounded or unconfounded at either time point.

*PANSS* positive and negative syndrome scale, *SHRS* St. Hans rating scale.

## Methods

### Study design and participants

Data from the EULAST were used for the present study ^53^.

EULAST was a pragmatic, randomized, open-label trial conducted at 50 general hospitals and psychiatric clinics in 15 European countries and Israel. Both inpatients and outpatients were recruited at the participating health-care facilities. Eligible participants were aged 18 years or older and met the DSM-IV criteria for schizophrenia as confirmed by the Mini International Neuropsychiatric Interview 5 plus (MINI-5)^54^ and had experienced their first psychotic episode from a minimum of 6 months to a maximum of 7 years before study entry. Participants were randomly allocated (1:1:1:1) using block randomization to LAI paliperidone, LAI aripiprazole, or to the respective oral formulations of these antipsychotics. Randomization was stratified by country and duration of illness (6 months up to 3 years vs 4–7 years). Participants were followed up for up to 19 months. The study was approved in each country by the respective regulatory authorities and ethics committees according to the local regulations and consistent with the Declaration of Helsinki. The University Medical Center Utrecht (Utrecht, the Netherlands) monitored the trial according to the Good Clinical Practice and International Conference on Harmonization guidelines^55^. Further details on study procedures are provided elsewhere ^53^.

The start of illness was defined by the first contact with a health-care professional in relation to psychotic symptoms. If participants were already using antipsychotics, a medication switch was to be under consideration by the treating physician. Exclusion criteria have been described in detail in the primary EULAST publication ^53^.

### Procedures

Within 10 days after the screening visit (visit 1; week −1), baseline visit (week 0) was conducted, and participants were randomly allocated to oral or long-acting formulations of aripiprazole or paliperidone. Assuming a steady state for all treatment groups at the end of the study drug titration period (4 weeks), participants underwent visit 4 assessments. Follow-up visits were scheduled after 12 (visit 15) and 18 (visit 21) months. Concomitant medications, including psychotropic drugs, were allowed as long as they were prescribed according to the local summaries of product characteristics (SmPC). Beyond visit 4, augmentation with a second antipsychotic was permitted up to a prespecified threshold; above this threshold, all-cause discontinuation criteria applied. The medications in the four treatment groups were dosed according to their respective SmPC; the medication dose was flexible throughout the trial and was at the clinician’s discretion.

#### Assessment

The Positive and Negative Syndrome Scale (PANSS) was used to assess psychopathology^56^. It is a hetero-administered clinical scale, which comprises 30 items grouped into three distinct subscales, one for positive symptoms (7 items), one for negative ones (7 items), and one for general psychopathological symptoms (16 items). Each item is rated on a seven-point Likert scale. Higher scores are associated with greater severity of symptoms. In the present study, PANSS was used to measure positive and negative dimensions, disorganization, and depression according to Wallwork et al^57^. In particular, the positive dimension was calculated by summing the scores on the items “delusions” (P1), “hallucinatory behavior” (P3), “grandiosity” (P5), and “unusual thought content” (G9); negative dimension was calculated by summing the scores on the items assessing core negative symptoms, i.e., “blunted affect” (N1), “emotional withdrawal” (N2), “poor rapport” (N3), “passive withdrawal” (N4) and “lack of spontaneity” (N6)^58^; the disorganization dimension was assessed using the item “conceptual disorganization” (P2); and the severity of depressive symptoms was assessed using the corresponding item (G6).

The St. Hans Rating Scale (SHRS) was used to assess extrapyramidal symptoms^59^. Specifically, the global parkinsonism score of the SHRS was employed to assess the absence of clinically relevant parkinsonism, a potential cause of secondary negative symptoms. Also, for SHRS, higher scores indicated more severe symptoms.

The Personal and Social Performance Scale (PSP) was used to assess functional outcome^60^. The PSP is a semi-structured interview that investigates four areas: self-care, socially useful activities, personal and social relationships, and disturbing and aggressive behaviors. For each area considered, a score from 0 (absent) to 5 (very severe) is assigned, with higher scores standing for worse functioning in that area. Out of the ratings on the four subdimensions, one total score on a 100-point scale can be created, with higher scores indicating better functioning.

### Classification of negative symptoms and definitions of study groups

The presence of negative symptoms of moderate severity was defined by a PANSS score >3 for at least one of the core negative symptoms as defined in the NIMH-MATRICS consensus conference^58^: blunted affect (N1), emotional withdrawal (N2), poor rapport (N3), passive withdrawal (N4), and lack of spontaneity (N6).

Participants with at least one of the core negative symptoms of moderate severity (NEG group) at the start of the study (baseline visit, week 0) were distinguished from participants who did not present any core negative symptoms of moderate severity (N-NEG group).

Unconfounded negative symptoms, in the present study, were defined according to the following criteria: (a) the presence of at least one negative symptom of moderate severity, and (b) the absence of both clinically significant depression (PANSS item G6 ≤ 3) and clinically significant parkinsonism—i.e., a “mild” (two) rating on at least three items, or a “mild” rating for tremor or rigidity plus a “mild” rating on at least another item, or a “mild-moderate (three or more) rating on at least one item of the Parkinsonism subscale of the SHRS. Therefore, within the NEG group, participants with unconfounded negative symptoms at week 0 (UNCONF group) were distinguished from participants with confounded negative symptoms (CONF group), who did not meet the *b* criterion specified above.

In the present paper, participants with persistent negative symptoms (PNS) are defined as those with unconfounded negative symptoms at baseline (week 0) that persisted and were not confounded by depression or parkinsonism also at visit 15 (1 year follow-up).

Conversely, non-persistent negative symptoms (N-PNS) were defined as the presence of at least one core negative symptom of moderate severity at baseline (either confounded or unconfounded), which did not persist at visit 15 (1 year follow-up).

Finally, enduring negative symptoms (E-NS) included negative symptoms that were either confounded or unconfounded at baseline (week 0) and persisted as either confounded or unconfounded at visit 15 (1-year follow-up).

Prospective consistency of PNS was defined as the percentage of participants with persistent negative symptoms at follow-up among those who exhibited unconfounded negative symptoms at baseline. The percentage was calculated after excluding individuals who dropped out before the 1-year follow-up (visit 15).

### Data analysis

Demographic and clinical variables recorded at baseline (week 0) were compared between the NEG and N-NEG groups. In addition, we compared the two groups on functioning total scores at baseline (week 0), visit 15 (12 months), and visit 21 (18 months), and drop-out rate at visits 15 and 21.

Differences at baseline between study completers and non-completers were examined, and logistic regression models were used to identify potential predictors of attrition.

PNS and N-PNS participants were compared on demographic and clinical variables at baseline (week 0), on drop-out rate after 18 months (visit 21), and on functioning at baseline, visit 15 (1 year), and visit 21 (18 months). Additionally, we compared participants with E-NS with those with N-PNS on the same variables.

To test group differences on categorical variables, the Pearson’s chi-square test was used; on continuous data, and depending on the number of groups considered, one-way analyses of variance (ANOVAs) and independent samples *t*-test were used. For the between-groups comparison of demographic and clinical variables at week 0 the significance level was adjusted according to Bonferroni, based on the number of tests in each set of analysis, and post-hoc pairwise comparisons were made when required. The between-groups comparison (NEG vs N-NEG, PNS vs N-PNS, and E-NS vs N-PNS) of functioning levels and drop-out rates was a confirmatory analysis, based on strong a priori evidence from previous studies^12,13,45,46,61–64^; therefore, no Bonferroni correction was applied in these analyses, and one-sided tests were used to increase statistical power in the hypothesized direction.

All statistical analyses were computed using SPSS Version 28.0 (IBM Corporation, 2021).

## Results

Demographic and clinical characteristics of participants included in the present study (*n* = 502) are reported in Table 2. Participants included in the study had a PANSS total score of 74.45 (SD = 18.38) and a total PSP score of 54.03 (SD = 16.88).

**Table 2:** Demographic characteristics and illness-related variables of the whole study sample at baseline (week 0).

Age (years, mean±SD)	*n* = 502	30.59 ± 9.68
Gender (males, %)	***n*** = **502**	335 (66.73%)
Education (years)	***n*** = **501**	11.91 ± 2.83
PANSS total score	***n*** = **502**	74.45 ± 18.38
PANSS positive	***n*** = **502**	10.76 ± 4.28
PANSS negative (core symptoms)	***n*** = **502**	14.47 ± 5.40
PANSS P2 (disorganization)	***n*** = **502**	2.59 ± 1.25
PSP - Socially useful activities (including work and study)	***n*** = **373**	3.58 ± 1.13
PSP - Personal and social relationships	***n*** = **373**	3.26 ± 1.15
PSP - Self-care	***n*** = **373**	2.03 ± 1.12
PSP - Disturbing and aggressive behaviors	***n*** = **373**	1.53 ± 0.96
PSP - Total score	***n*** = **372**	54.03 ± 16.88
Global Parkinsonism	***n*** = **370**	0.39 ± 0.99
Study Medication (aripiprazole/paliperidone)	***n*** = **500**	*n* = 245/*n* = 255

*PANSS* positive and negative syndrome scale, *PSP* Personal and Social Performance scale (for each domain of functionin, higher scores stand for worse functioning; for the total score: higher score stands for better functioning), *SD* standard deviation.

### Prevalence, characterization, and persistence of negative symptoms

**Fig. 1:**
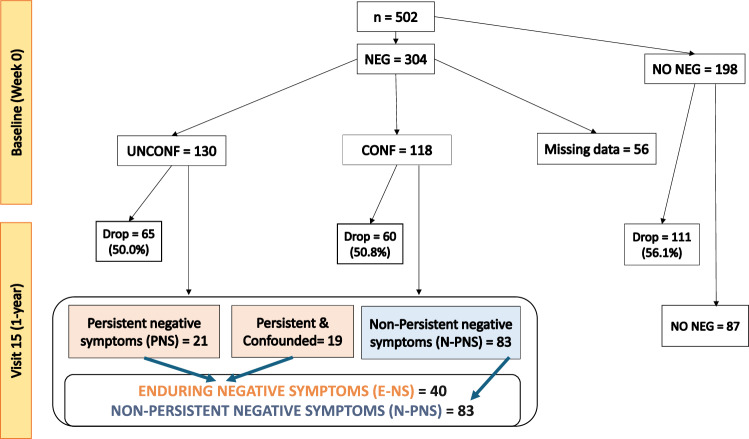
Number of participants in the study for each category. *NEG group:* participants with at least one of the core negative symptoms of moderate severity at baseline; *N-NEG group:* participants with no core negative symptoms of moderate severity at baseline; *UNCONF group:* participants with at least one negative symptom of moderate severity and no clinically significant depression at baseline; *CONF group:* participants with at least one negative symptom of moderate severity but clinically significant depression and/or parkinsonism at baseline; *PNS group:* participants with unconfounded negative symptoms at baseline that persisted and were not confounded by depression or parkinsonism after one year of follow-up; *N-PNS group:* participants with at least one core negative symptom of moderate severity at baseline (whether confounded or unconfounded), which either did not persist after one year or persisted but was confounded by clinically significant depression or parkinsonism after one year; *E-NS group:* participants that presented either PNS after one year of follow-up or confounded negative symptoms at baseline that persisted after 1 year.

Three-hundred and four participants (60.6% of the study sample) had a PANSS score >3 for at least one negative symptom at the baseline (week 0) and were included in the NEG group (Fig. 1). Within this group, 130 participants (42.8%) showed unconfounded negative symptoms of moderate severity (NEG UNCONF group), while 118 participants (38.8%) presented confounded negative symptoms (NEG CONF group). Fifty-six participants (18.4%) could not be categorized within the UNCONF or CONF group since their data relevant to parkinsonism was missing (Fig. 1).

After 1-year (visit 15), out of the 130 NEG UNCONF, 118 NEG CONF, and 198 NO NEG participants, respectively 65 (50%), 60 (50.8%) and 111 (56.1%) dropped-out from the study. The drop-out rates did not differ among the three groups. Among the remaining participants at visit 15 (12 months), the frequency of PNS was 7.9% (21/266). Excluding drop-out participants, the prospective consistency of PNS at 1 year follow-up was 32.3% (21/65). More specifically, the symptom that more frequently persisted after 1 year was blunted affect (12/21), followed by emotional withdrawal (11/21), and passive withdrawal (8/21). Out of the NEG group, eighty-three participants had no PNS after 1 year (N-PNS group); 19 participants had persistent negative symptoms at one year, but those symptoms were confounded at either baseline (week 0) or visit 15 (12 months). These 19 participants together with those in the PNS group (21 participants), were classified as participants with enduring negative symptoms (E-NS group, *n* = 40). The frequency of E-NS at 1 year follow-up was 15% (40/266), and the prospective consistency was 32.5% (40/123) (Fig. 1).

### Sociodemographic, clinical, and psychosocial functioning variables: comparisons between NEG and N-NEG participants at baseline (week 0)

Group comparisons on sociodemographic and clinical variables, as recorded at baseline (week 0), are reported in Table 3. The NEG group displayed a significantly higher PANSS total score (*p* < 0.001), greater severity of negative symptoms (*p* < 0.001), disorganization (*t* = 2.906; *p* = 0.004), and depression (*p* < 0.001) (Table 3). A trend-level effect was observed for parkinsonism, since the NEG group displayed higher scores on global parkinsonism, (*p* = 0.017), but the difference did not survive the correction for multiple testing.

**Table 3:** Sociodemographic and illness-related variables at baseline (week 0): comparisons between NEG and N-NEG groups.

	NEG (*n* = 304)	N-NEG (*n* = 198)	*t*/*χ*^2^	*p*
	Mean±SD (*n*)			
Age (years)	30.38 ± 9.79 (*n* = 304)	30.90 ± 9.53 (*n* = 198)	0.591	0.555
Gender (M/F)	211/93 [69.4% males]	124/74 [62.6% males]	2.484	0.115
Education (years)	11.84 ± 2.74 (*n* = 304)	12.02 ± 2.98 (*n* = 197)	0.681	0.496
PANSS total score	81.21 ± 17.10 (*n* = 304)	64.07 ± 15.21 (*n* = 198)	11.458	<0.001*
PANSS positive	11.02 ± 4.38 (*n* = 304)	10.38 ± 4.11 (*n* = 198)	1.634	0.103
PANSS negative (core symptoms)	17.63 ± 4.12 (*n* = 304)	9.61 ± 2.97 (*n* = 198)	23.656	<0.001*
PANSS P2 (disorganization)	2.72 ± 1.27 (*n* = 304)	2.39 ± 1.19 (*n* = 198)	2.906	0.004*
PANSS G6 (depression)	2.81 ± 1.43 (*n* = 304)	2.31 ± 1.23 (*n* = 198)	4.056	<0.001*
Global Parkinsonism	0.49 ± 1.10 (*n* = 220)	0.24 ± 0.77 (*n* = 149)	2.398	0.017*
Study Medication (aripiprazole/paliperidone)	154/150 (*n* = 304)	91/105 (*n* = 196)	0.652	0.419

*NEG group:* participants with at least one of the core negative symptoms of moderate severity at baseline.

*N-NEG group:* participants with no core negative symptoms of moderate severity at baseline.

*PANSS* positive and negative syndrome scale, *SD* standard deviation.

*p < 0.05; **in bold statistically significant results** (Bonferroni-adjusted p-value: *p* < 0.005).

Furthermore, participants in the NEG group displayed significantly worse functioning at baseline *p* < 0.001), as compared to participants of the N-NEG group (Table 4). Differences in each functioning area were explored at baseline. The NEG group showed worse functioning than N-NEG (*p* < 0.05) in all PSP domains except one (disturbing and aggressive behaviors).

**Table 4:** Functional outcome and drop-out rate at different time points: Comparisons between NEG and N-NEG groups.

PSP total score	NEG (*n* = 304)	N-NEG (*n* = 198)	*t*	*p*	Cohen’s *d*	Lower CI	Upper CI
	Mean ± SD (*n*)						
Baseline (week 0)	50.97 ± 16.13 (*n* = 233)	59.40 ± 16.75 (*n* = 138)	4.794	<0.001*	−0.515	−0.728	−0.301
Visit 15 (12 months)	63.11 ± 18.92 (*n* = 80)	66.25 ± 16.21 (*n* = 44)	0.928	0.178	−0.174	−0.542	0.195
Visit 21 (18 months)	61.54 ± 18.29 (*n* = 57)	67.06 ± 15.28 (*n* = 33)	1.462	0.074	−0.320	−0.750	0.112
**Drop-out**	**Drop**^**a**^**/total (percentage)**		***χ***^2^	***p***	**Cramér’s V**	**Lower CI**	**Upper CI**
Visit 15 (12 months)	150/304 (49.3%)	111/198 (56.1%)	2.168	0.141	0.066	0.004	0.154
Visit 21 (18 months)	160/304 (52.6%)	117/198 (59.1%)	2.023	0.155	0.063	0.004	0.151

*NEG group:* participants with at least one of the core negative symptoms of moderate severity at baseline.

*N-NEG group:* participants with no core negative symptoms of moderate severity at baseline.

*p < 0.05.

*PSP* Personal and Social Performance scale (higher score stands for better functioning).

^a^Number of participants who dropped out from the study before completing the specified visit.

No statistically significant differences in functioning were recorded between NEG and N-NEG at the 12 (visit 15) and 18 months (visit 21) follow-up evaluations. Finally, no statistically significant differences between NEG and N-NEG emerged in drop-out rates at the two follow-up evaluations (Table 4).

### Sociodemographic, clinical, and functioning variables: comparisons between PNS and N-PNS participants

At baseline (week 0), PNS and N-PNS participants differed on depression severity: the N-PNS group exhibited higher severity of depression (*p* < 0.001) (Table 5). A significantly higher percentage of PNS patients were allocated to aripiprazole (15/21, 71.4%) than N-PNS patients (37/83, 44.6%). The two groups did not differ on other sociodemographic or illness-related variables at week 0 (Table 5).

**Table 5:** Sociodemographic and illness-related variables at baseline (week 0): comparisons between PNS and N-PNS.

	PNS (*n* = 21)	N-PNS (*n* = 83)	*t*/*χ*^2^	*p*
	Mean ± SD (*n*)			
Age (years)	31.00 ± 11.54 (*n* = 21)	28.99 ± 9.07 (*n* = 83)	0.858	0.393
Gender (M/F)	16/5 [76.2% males]	54/29 [65.1% males]	0.944	0.331
Education (years)	13.19 ± 4.27 (*n* = 21)	11.57 ± 2.86 (*n* = 83)	2.085	0.040*
PANSS total score	71.95 ± 13.69 (*n* = 21)	78.02 ± 15.04 (*n* = 83)	1.681	0.096
PANSS positive	9.48 ± 3.57 (*n* = 21)	10.18 ± 4.38 (*n* = 83)	0.680	0.498
PANSS negative (core symptoms)	17.57 ± 3.21 (*n* = 21)	16.43 ± 23.28 (*n* = 83)	1.422	0.158
PANSS P2 (disorganization)	2.24 ± 1.22 (*n* = 21)	2.53 ± 1.14 (*n* = 83)	1.034	0.304
PANSS G6 (depression)	1.67 ± 0.79 (*n* = 21)	2.94 ± 1.40 (*n* = 83)	3.975	<0.001*
Global Parkinsonism	0.10 ± 0.43 (*n* = 21)	0.34 ± 0.86 (*n* = 71)	1.738	0.087
Study Medication(aripiprazole/paliperidone)	15/6	37/46	4.833	0.028*

*PNS group:* participants with unconfounded negative symptoms at baseline that persisted and were not confounded by depression or parkinsonism at Visit 15;

*N-PNS group:* participants with at least one core negative symptom of moderate severity at baseline (whether confounded or unconfounded), which either did not persist at Visit 15 or persisted but was confounded by clinically significant depression or parkinsonism at Visit 15.

*p < 0.05; **in bold statistically significant results** (Bonferroni-adjusted p-value: *p* < 0.005).

*PANSS* positive and negative syndrome scale, *SD* standard deviation.

In terms of functioning, the two groups were comparable at week 0 (*t* = 0.875, *p* = 0.194), but PNS participants showed significantly worse functioning at 12 (*t* = 1.966, *p* = 0.028) and 18 (*t* = 2.036, *p* = 0.024) months of follow-up (Table 6). No significant differences were observed between PNS and N-PNS participants in terms of drop-out rates (Table 6).

**Table 6:** Functioning outcome and drop-out rate at different time points: Comparisons between PNS and N-PNS groups.

PSP total score	PNS (*n* = 21)	N-PNS (*n* = 83)	*t*	*p*	Cohen’s d	Lower CI	Upper CI
	Mean±SD (n)						
Baseline (week 0)	51.00 + 9.33	53.97 + 17.81	0.875	0.194	-0.179	-0.761	0.404
Visit 15 (12 months)	55.50 + 15.71 (*n* = 10)	67.77 + 18.02 (*n* = 39)	1.966	0.028*	-0.697	-1.402	0.016
Visit 21 (18 months)	54.23 + 15.39 (*n* = 13)	66.53 + 19.24 (*n* = 30)	2.036	0.024*	-0.676	-1.339	-0.005
**Drop-out**	**Drop**^**a**^**/total (percentage)**		**χ2**	**p**	**Cramér’s V**	**Lower CI**	**Upper CI**
Visit 21 (18 months)	1/21 (4.8%)	6/83 (7.2%)	0.162	0.687	0.040	0.004	0.175

*PNS group:* participants with unconfounded negative symptoms at baseline that persisted and were not confounded by depression or Parkinsonism at Visit 15.

*N-PNS group:* participants with at least one core negative symptom of moderate severity at baseline (whether confounded or unconfounded), which either did not persist at Visit 15 or persisted but was confounded by clinically significant depression or Parkinsonism at Visit 15.

*p < 0.05.

*PSP* Personal and Social Performance scale (higher score stands for better functioning).

^a^Number of participants who dropped out from the study before completing the specified visit.

### Sociodemographic, clinical, and functioning variables: comparisons between participants with (E-NS) and without (N-PNS) enduring negative symptoms

Group comparisons for demographic and clinical characteristics between E-NS and N-PNS are reported in Table 7. As expected, the E-NS participants showed a higher severity of negative symptoms at week 0 (*p* < 0.001). The two groups were comparable for all the other explored variables (Table 7). In terms of functioning, the two groups were comparable at week 0 (*t* = 1.131, *p* = 0.130), but E-NS participants showed significantly worse functioning at 12 (*t* = 3.274, *p* < 0.001) and 18 (*t* = 2.516, *p* = 0.008) months of follow-up (Table 8). No significant differences were observed between E-NS and N-PNS participants in terms of drop-out rates (Table 8).

**Table 7:** Differences between E-NS and N-PNS groups in sociodemographic and illness-related variables at baseline (week 0).

	E-NS (*n* = 40)	N-PNS (*n* = 83)	*t*/*χ*^2^	*p*
	Mean ± SD (*n*)			
Age (years)	32.38 ± 10.42 (*n* = 40)	28.99 ± 9.07	1.847	0.067
Gender (F/M)	29/11	54/29	0.681	0.409
Education (years)	12.95 ± 3.41 (*n* = 40)	11.57 ± 2.86 (*n* = 83)	2.354	0.020*
PANSS total score	78.20 ± 18.39 (*n* = 40)	78.02 ± 15.04 (*n* = 83)	0.056	0.955
PANSS positive	9.20 ± 3.89 (*n* = 40)	10.18 ± 4.38 (*n* = 83)	1.204	0.231
PANSS negative (core symptoms)	19.08 ± 4.05 (*n* = 40)	16.43 ± 23.28 (*n* = 83)	3.863	<0.001*
PANSS P2 (disorganization)	2.35 ± 1.18 (*n* = 40)	2.53 ± 1.14 (*n* = 83)	0.809	0.420
PANSS G6 (depression)	2.78 ± 1.83 (*n* = 40)	2.94 ± 1.40 (*n* = 83)	0.549	0.584
Global Parkinsonism	0.54 ± 1.19 (*n* = 37)	0.34 ± 0.86 (*n* = 71)	1.013	0.313
Study medication (aripiprazole/paliperidone)	20/20	37/46	0.319	0.572

*E-NS group:* participants that presented either PNS at visit 15 or confounded negative symptoms at baseline that persisted at visit 15.

*N-PNS group:* participants with at least one core negative symptom of moderate severity at baseline (whether confounded or unconfounded), which either did not persist at visit 15 or persisted but was confounded by clinically significant depression or parkinsonism at visit 15.

*p < 0.05; **in bold statistically significant results** (Bonferroni-adjusted p-value: *p* < 0.005).

*PANSS* positive and negative syndrome scale, *SD* standard deviation.

**Table 8:** Comparisons between E-NS and N-PNS groups in functioning outcome and drop-out rate at different time points.

PSP total score	E-NS (*n* = 40)	N-PNS (*n* = 83)	*t*	*p*	Cohen’s d	Lower CI	Upper CI
	Mean ± SD (*n*)						
Baseline (week 0)	49.76 ± 13.10 (*N* = 29)	53.97 ± 17.81 (*N* = 60)	1.131	0.130	−0.256	−0.700	0.190
Visit 15 (12 months)	52.57 ± 17.02 (*N* = 23)	67.77 ± 18.03 (*N* = 39)	3.274	<0.001*	-0.861	−1.395	−0.320
Visit 21 (18 months)	53.26 ± 15.77 (*N* = 19)	66.53 ± 19.24 (*N* = 30)	2.516	0.008*	−0.738	−1.327	−0.140
**Drop-out**	**Drop**^**a**^**/total (percentage)**		***χ***^2^	***p***	**Cramér’s V**	**Lower CI**	**Upper CI**
Visit 21 (18 months)	3/40 (7.5%)	6/83 (7.2%)	0.003	0.957	0.005	0.004	0.200

*E-NS group:* participants who presented either PNS at visit 15 or confounded negative symptoms at baseline that persisted at visit 15.

*N-PNS group:* participants with at least one core negative symptom of moderate severity at baseline (whether confounded or unconfounded), which either did not persist at visit 15 or persisted but was confounded by clinically significant depression or Parkinsonism at visit 15.

*p < 0.05.

*PSP* Personal and Social Performance scale (higher score stands for better functioning).

^a^Number of participants who dropped out from the study before completing the specified visit.

## Discussion

In this post-hoc analysis, we examined the prevalence and impact on functional outcome of the long-term persistence of negative symptoms in a large group of participants in the early phase of schizophrenia.

To ensure accuracy in assessing negative symptoms, we focused on five core negative symptoms and identified negative symptoms unconfounded by depression and extrapyramidal symptoms. In line with previous research conducted on participants in the early stages of the disorder,^13,43,45,48^, we did not set a threshold for the severity of positive symptoms and disorganization to define PNS, as done in previous studies on first-episode participants^14,15^ given that the presence of active psychotic symptoms was required to enter the main study.

According to our data, at baseline, 304 (60.6%) out of the 502 early-phase psychotic participants enrolled in the study had at least one negative symptom of moderate severity. Among them, 130 (42.8%) exhibited negative symptoms that were not confounded by depression or Parkinsonism. After 1 year of follow-up, excluding participants who dropped out from the trial, unconfounded negative symptoms persisted in 21 out of 266 (7.9%) individuals. Attrition analyses indicated that participants who discontinued the study did not differ from completers in baseline functioning or negative symptom severity (Tables S1–S2). However, non-completers exhibited more severe positive symptoms at baseline, and higher baseline depressive symptoms significantly predicted drop-out at 18 months (Tables S1–S2). The reference group (*n* = 266) included all participants with negative symptoms at baseline and available follow-up data to determine symptom persistence, including those with missing information only on depression or Parkinsonism. These participants were categorized as Persistent Negative Symptoms (PNS). Previous research conducted in individuals in the early-phase of psychosis has indicated a prevalence of PNS ranging from 4 to 41%^5,12,13,41–50^. As stated in the introduction, the variability in prevalence rates among different studies is mainly determined by the criteria used for defining PNS. First, the number of negative symptoms of moderate severity that should persist across time to identify participants with PNS varies among different studies,^13,43,47,48^, and several works even employ symptoms that did not consistently align with negative symptom factors^41,42,46^. Moreover, some studies considered depression and/or Parkinsonism as confounding factors for negative symptoms,^13,43,45,48^, others confounded only depression,^46^, while others additionally accounted for positive symptoms and disorganization^5,47,48^. Finally, the definition of persistence is largely influenced by the differences in follow-up duration, ranging from 3 months to 3 years in studies involving first-episode participants.

In our study, among the 65 participants who had unconfounded negative symptoms at baseline and completed the 1-year follow-up, 21 (32.3%) continued to exhibit unconfounded negative symptoms, representing the prospective consistency of unconfounded negative symptoms in our sample. This is almost in line with the findings of a previous multicenter research using similar inclusion and exclusion criteria, which reported a consistency of around 20%^13^. That study also investigated the impact of the unconfounded negative symptoms persistence on functional outcome in a large sample of participants who had experienced the onset of psychotic symptoms within two years before the study recruitment^13^. The results of that study showed less global functioning improvement from baseline to follow-up in PNS participants as compared to N-PNS ones. The present analysis confirmed those findings in an independent sample comprising participants with schizophrenia spectrum disorders who had experienced illness onset within the previous seven years. Specifically, we observed that, although participants with PNS showed similar levels of functional impairment at baseline as compared to those with N-PNS, they demonstrated very limited improvement in functioning after 12 and 18 months of treatment, regardless of the treatment arm to which they had been randomized. The replication of these results in two recent-onset schizophrenia cohorts with different illness durations reinforces the notion that the long-term persistence of primary negative symptoms adversely affects functional outcomes independently of the disease stage, and appears largely unresponsive to standard pharmacological interventions^12,13,45,46,61–65^. Performing an additional analysis comparing individuals with enduring negative symptoms (E-NS)—a broader category including both confounded and unconfounded persistent negative symptoms—with N-PNS participants, the association with functional impairment showed a larger effect size. Indeed, including cases with symptoms confounded by depression or Parkinsonism yielded numerically larger effect sizes for the E-NS vs N-PNS comparison at 12 and 18 months; however, the overlapping 95% confidence intervals do not support a statistical difference between these effect sizes. This larger point estimates may partly reflect differences in sample size or group balance; in fact, the comparison group expanded from 21 individuals in the PNS group to 40 in the E-NS group, adding cases with persistent and confounded negative symptoms at the 1-year follow-up, while control group (N-PNS) remained constant (*N* = 83), The prevalence of E-NS was nearly twice that of N-PNS (15% vs. 7.9%), although the prospective consistency remained stable at around 32%.

These findings suggest that, from a pragmatic clinical perspective, persistent negative symptoms—regardless of whether they are unconfounded—may represent a useful and clinically meaningful indicator of poor long-term psychosocial functioning. In other words, even when the primary or secondary nature of negative symptoms cannot be fully determined, their persistence over time still carries prognostic value and may help to identify individuals with poorer functional outcomes. This has important implications for both clinical practice and research. From a clinical perspective, it supports the consideration of negative symptom persistence as a clinically informative indicator for early detection, monitoring, and stratification of patients who may require tailored psychosocial or rehabilitative interventions. From a clinical perspective, it supports the consideration of negative symptom persistence as a clinically informative indicator of the need for early detection, monitoring, and stratification of patients who may require tailored psychosocial or rehabilitative interventions. From a research perspective, it highlights the need to complement the distinction between primary and secondary negative symptoms with a focus on their temporal stability and functional significance. Accordingly, persistent negative symptoms should be regarded as a core treatment target and a meaningful outcome for future intervention studies.

Future models of early intervention might benefit from incorporating a more inclusive definition, recognizing that depressive or extrapyramidal symptoms often co-occur and may interfere with functional performance in real-world settings.

## Study limitations

This study presents some limitations. First, the prevalence of PNS may have been underestimated due to dropout and missing data at follow-up assessments. Second, the use of the PANSS to assess negative symptoms is an additional limitation, given its poor assessment of avolition, the negative symptom with the highest impact on functioning^2,15,17,37,66^. Furthermore, a substantial proportion of participants lacked the PSP functional assessment, which further reduced the sample size in the comparative analyses between the two groups, potentially limiting the statistical power to detect significant differences in functioning. Another limitation concerns the variability in illness duration. Although all participants were in the early course of schizophrenia spectrum disorders, the inclusion of patients up to seven years post-onset might have introduced significant sample heterogeneity.

## Conclusions

In conclusion, our findings confirm that negative symptoms that persist across time represent a clinically relevant dimension in early-phase schizophrenia. Indeed, the long-term persistence of these symptoms, independently of the baseline functioning levels and their primary or secondary nature, seems to contribute to the poor functional outcome of participants with schizophrenia. These results highlight the prognostic value of persistent or enduring negative symptoms and underscore their relevance as an early target for monitoring, treatment planning, and intervention.

Future research should further investigate predictors of symptom persistence to guide the development of targeted interventions aimed at improving functional outcomes in this population.

## Supplementary information

EULAST PNS_Supplementary materials

## Data Availability

The data that support the findings of this study cannot be made publicly available for confidentiality reasons. However, data are available from the corresponding author upon reasonable request.
