# Supplementary material for: Persistent negative symptoms in the EULAST cohort: impact on functional outcome
Source: Schizophrenia (Heidelb). 2026 Mar 2;12(1):36. doi: 10.1038/s41537-026-00739-w (PMC13065952; doi:10.1038/s41537-026-00739-w)
Supplement: Supplementary file 1 — EULAST PNS_Supplementary materials [file 41537_2026_739_MOESM1_ESM.pdf]

Supplementary Material

Title: Persistent negative symptoms in the EULAST cohort: relevance to functional outcome

Results

1. Attrition analysis: comparison between completers and non-completers at 18 months.
2. The effects of study medication allocation on the course of negative symptoms severity

Tables and figures

Table S1. Sociodemographic and illness-related variables at baseline (week 0): comparison between patients who completed the study and those who dropped out at 18 months

Table S2. Logistic regression model examining baseline predictors of 18-month study discontinuation

Figure S1. Negative symptoms severity in Aripiprazole and Paliperidone study groups across study visits

Results

1 Attrition analysis: comparison between completers and non-completers at 18 months.

Baseline comparisons between completers and non-completers showed no significant differences in sociodemographic characteristics or in most illness-related variables, including negative symptoms, disorganization, parkinsonism, and other PSP functional domains. However, participants who discontinued the study had significantly higher baseline positive symptom severity ($t = -2.951$, $p = .003$) and slightly higher depressive symptoms ($t = -2.001$, $p = .046$). In addition, a marginal difference was observed in the PSP disturbing and aggressive behaviors domain ($t = -2.007$, $p = .046$). These findings suggest that greater baseline severity of positive and, to a lesser extent, depressive symptoms, as well as mildly higher levels of disturbing behaviors, may be associated with an increased likelihood of study discontinuation, whereas negative symptoms and overall functioning did not appear to substantially influence attrition (Table S3).

2 The effects of study medication allocation on the course of negative symptoms severity

As an exploratory analysis, given that the PNS and N-PNS groups showed a trend-level effect regarding medication allocation, a repeated measures ANOVA with a between-subjects factor (medication) was conducted to assess differences in the improvement of negative symptom severity over time between the two groups (Figure S1). The analysis showed no significant interaction between group (medication allocation) and time ($F_{(2.28,\ 439.25)}=32.831$, $p=0.076$). The results indicated a significant main effect of time ($F_{(2.28,\ 439.25)} = 49.692$, $p<0.001$) and post-hoc analysis showed that negative symptoms severity decreased at visit 15 and 21, as compared to both baseline and visit 4, for both groups ($p<0.05$). Finally, no significant group effect was recorded ($F_{(1,\ 192)} = 3.078$, $p = 0.081$), but subjects assigned to the paliperidone treatment reported higher negative symptoms severity at both baseline ($p=0.022$) and visit 4 ($p=0.035$), as compared to the aripiprazole group at the corresponding time points (Figure S1).

Table S1. Sociodemographic and illness-related variables at baseline (week 0): comparison between patients who completed the study and those who dropped out at 18 months

	No Drop-out (n=225)	Drop-out (n=277)	t/χ2	p
	(Mean±SD)			
**Age (years)**	30.85±9.82	30.37±9.57	0.552	.581
**Gender (F/M)**	80/145	87/190	0.962	.327
**Education (years)**	12.06±3.05	11.78±2.64	1.080	.281
**PANSS positive**	10.14±4.32	11.27±4.18	-2.951	.003*
**PANSS negative (core symptoms)**	14.42±5.32	14.51±5.48	-0.172	.863
**PANSS P2 (disorganization)**	2.51±1.48	2.65±1.30	-1.254	.210
**PANSS G6 (depression)**	2.48±1.39	2.72±1.36	-2.001	.046
**Global Parkinsonism**	0.36±0.95	0.41±1.01	-0.518	.605
**PSP - Socially useful activities**	3.46±1.18	3.68±1.09	-1.826	.069
**PSP - Personal and social relationships**	3.18±1.11	3.33±1.10	-1.154	.249
**PSP - Self-care**	2.01±1.11	2.03±1.11	-0.418	.676
**PSP - Disturbing & aggressive behaviors**	1.75±0.84	1.61±1.03	-2.007	.046
**Study Medication (aripiprazole/paliperidone)**	115/110	130/145	0.730	.393

PANSS = Positive and Negative Syndrome Scale; PSP = Personal and Social Performance scale; SD = standard deviation

* statistically significant difference after Bonferroni's correction for multiple tests

Table S2. Logistic regression model examining baseline predictors of 18-month study discontinuation

	B	S.E.	p
**Age**	-0.005	0.015	0.731
**Gender**	0.095	0.286	0.740
**Education**	-0.025	0.055	0.648
**PANSS positive**	0.008	0.032	0.790
**PANSS negative (core symptoms)**	-0.009	0.028	0.734
**PANSS P2 (disorganization)**	0.076	0.104	0.465
**PANSS G6 (depression)**	0.270	0.102	0.008*
**Global Parkinsonism**	0.198	0.162	0.223
**PSP - Socially useful activities**	-0.072	0.163	0.660
**PSP - Personal and social relationships**	0.017	0.134	0.899
**PSP - Self-care**	0.113	0.162	0.484
**PSP - Disturbing & aggressive behaviors**	-0.014	0.139	0.918
**Study Medication**	0.152	0.264	0.566

PANSS = Positive and Negative Syndrome Scale; PSP = Personal and Social Performance scale; SD = standard deviation

* statistically significant difference

Figure S1. Negative symptoms severity in Aripiprazole and Paliperidone study groups across study visits

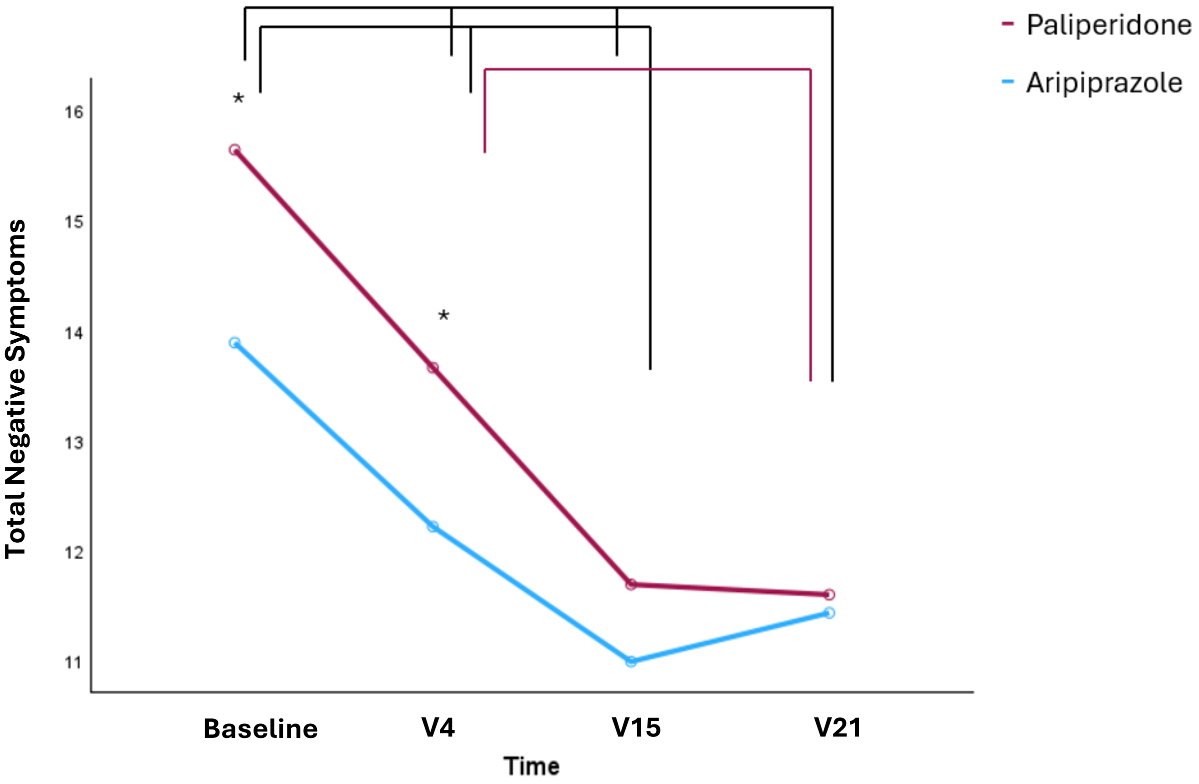

Baseline (Week 0); V4: Visit 4 (1-month follow up); V15: Visit 15 (1-year follow up); V21: Visit 21 (18-months follow up)

Significant differences in negative symptoms severity between different time points within each medication group ($p < 0.05$) were highlighted by lines (in both groups: V21 < Baseline & V4 while V15 < Baseline & V4; only in the paliperidone group: V21 < V4), while differences in negative symptoms severity between groups at the same time point were flagged by an asterisk (aripiprazole < paliperidone both at Baseline & V4)
